# A Quarter of a Million for Extensions and Improvements

**Published:** 1920-07-31

**Authors:** 


### RED CROSS SURPLUS FUNDS.

### A Quarter of a Million for Extensions and Improvements.

### KING EDWARD'S HOSPITAL FUND DISTRIBUTION.

A special meeting of the President and General Council of King Edward's Hospital Fund for London was held at St. James's Palace on July 23 to distribute the sum of £250,000 entrusted to the Fund by the Joint Committee of the British Red Cross Society and the Order of St. John of Jerusalem for distribution on their behalf in aid of schemes of extension or improvement at London hospitals able and willing to treat ex-sailors or ex-soldiers. The Viscount Finlay (member of the President's Powers Committee) presided.

There were present: The Governor of the Bank of England (Mr. M. C. Norman, member of the President's Powers Committee), the Chief Rabbi (the Rev. J. H. Hertz, D.D.), the Earl of Iveagh, the Viscount Mersey, the Viscountess Harcourt, the Lord Stamfordham, the Lord Somerleyton (Hon. Secretary), the Lord Stuart of Wortley, the President of the Hospital Sunday and Hospital Saturday Funds (the Rt. Hon. the Lord Mayor), the Chairman of the County Council (Mr. John W. Gilbert), the President of the Royal College of Physicians (Sir Norman Moore, Bt.), the President of the Royal College of Surgeons (Sir Anthony Bowlby), the Rt. Hon. Sir Horace Marshall, Sir William Church, Bt., Sir George Makins, Sir William H. Bennett, Sir William J. Collins, Sir Frederick M. Fry (Hon. Secretary), Sir John Craggs, Sir John Tweedy, Mr. John G. Griffiths (Hon. Secretary), Mr. Yvon R. Eccles.

**Lord Somerleyton** (Hon. Secretary) read the minutes of the last meeting, which were confirmed, and reported letters of regret.

**Mr. John G. Griffiths** (Hon. Secretary) reported the appointment of Sir George Makins to the General Council.

### DISTRIBUTION COMMITTEE'S REPORT.

The Chairman of the Distribution Committee (**Sir John Tweedy**) presented the Report of the Committee as follows:—

1. In July 1919 the Joint Finance Committee of the British Red Cross Society and the Order of St. John of Jerusalem invited the King's Fund to undertake on their behalf the distribution of £250,000 out of the surplus funds at the disposal of the Joint Committee amongst hospitals in the area of the Fund, upon conditions similar to those on which the Joint Committee had already made a distribution of surplus funds amongst hospitals outside London. The Joint Committee wished the distribution to be kept distinct from the ordinary distribution which is made in December. A preliminary notice was accordingly issued by the Distribution Committee in the autumn of 1919, stating that applications would not be actually invited until the early part of 1920.

Matured Schemes, with Time Limit.

2. The conditions provided, *inter alia*—

(*a*) that the distribution should be confined to hospitals in the London area that could and would treat ex-sailors and ex-soldiers, but that, amongst such hospitals, the distribution should not necessarily be subject to the whole of the conditions ordinarily attaching to grants made by the King's Fund out of its general funds;

(*b*) that the distribution should take the form of capital grants in aid of definite schemes of extension or improvement that would directly or indirectly increase the facilities for the treatment of ex-Service men, no part of it being available for maintenance or for the payment of debts;

(*c*) that before any grant was actually paid over the scheme must be mature. This did not mean that building must have begun, but that the scheme must be finally settled in the form in which it would be carried out; the plans, for example, having been passed by the Hospital Committee, the King's Fund, and any public or other authorities to whom the scheme required to be submitted;

(*d*) that before any grant was paid over the Hospital Committee should have received, in the form of contributions to the actual scheme, a sum equal to the grant or to the balance of the cost, whichever might be the smaller;

(*e*) that there should be a time limit for the fulfilment of these last two conditions, the aim being that all the grants should be paid over by March 31, 1921.

For Treatment of Ex-Service Men.

3. As the result of the first of these conditions this special distribution excludes certain hospitals (*e.g.*, hospitals for women and children only) which are eligible for the Fund's ordinary distribution and includes some hospitals which are ineligible under the ordinary rules. Inclusion or exclusion at this special distribution is, therefore, no indication of the attitude of the Distribution Committee at the ordinary distribution.

4. Applications, not *prima facie* ineligible for consideration, were received from sixty-four hospitals in respect of eighty-one schemes. Grants are recommended to forty-nine hospitals in respect of fifty-four schemes.

5. It must not be assumed that the absence of a grant implies dissatisfaction. In some cases, for instance, the scheme was too small to need a special grant, in others there was no prospect of fulfilling the condition as to maturity or as to the raising of funds, while at some hospitals a grant is recommended to only one scheme out of several submitted.

The Fund's Proviso.

6. Wherever a grant is recommended in aid of any scheme involving substantial structural additions or improvements, the hospital has been visited by a medical member of the Distribution Committee and either a lay member or one of the Honorary Secretaries, and the proposals, with sketch plans and estimates, have been discussed with the hospital representatives after an inspection of the existing accommodation and of the site. Wherever a scheme has been passed by the Distribution Committee the decision has been subject to the proviso that the Hospital Committee are satisfied as to the prospect of the provision of funds, both for the capital expenditure involved and for the subsequent cost of maintenance.

Where the proposals are not yet sufficiently mature to be passed in detail by the Committee, the grant will be subject to the further particulars of the scheme being submitted when ready.

Bed Extension not Encouraged.

7. In making this distribution in aid of schemes of capital expenditure, the Committee have taken into account the financial position of each hospital, and they have particularly borne in mind the importance of not unduly encouraging the provision of additional beds. They announced at the outset that in the case of schemes of bed extension they would require specially full and detailed replies to their usual inquiries as to the reasons for the proposal and as to the prospect of funds for future maintenance. After discussion with representatives of the Joint Committee of the British Red Cross Society and the Order of St. John of Jerusalem, it was ultimately decided that much the larger proportion of the £250,000 should be devoted to improve-

**Red Cross Surplus Funds**—(*continued*).

ments which would involve only comparatively small additional expenditure on maintenance.

General Survey of Extension Schemes.

8. In order to determine the distribution even of the smaller sum allocated to the provision of additional beds, it became necessary to devote considerable attention to the general survey of extension schemes, to which reference has been made in the last two annual reports of the Distribution Committee. The figures given in the report of last December showed 2,927 additional beds "definitely contemplated," and 1,144 "indefinite or future." The totals now amount to 3,445 and 2,081 respectively. These figures include hospitals ineligible for consideration under the conditions of this special distribution, as well as those eligible. As the result of the progress already made with the general survey, the schemes can now be sub-divided as follows:

Additional beds passed by the Distribution Committee (subject to the hospital authorities being satisfied as to the prospect of funds for building and maintenance), 1,489.

Additional beds which the Distribution Committee are not prepared to entertain or have referred back for reconsideration, 1,188.

Additional beds deferred at the instance of the Fund and to be re-submitted later, 429.

Additional beds, no opinion expressed, 339.

Additional beds indefinite or future, 2,081.

9. The bed extensions passed by the Fund, whether eligible for Red Cross grants or not, may be further subdivided as follows, according to the class of hospital and the geographical situation:

| | Additional Beds at General Hospitals or Cottage Hospitals | Additional Beds at Special Hospitals | Total |
|---|---|---|---|
| Within 3 miles of Charing Cross | 257 | 319 | 576 |
| From 3 to 6 miles of Charing Cross | 291 | 22 | 313 |
| From 6 to 9 miles of Charing Cross | 321 | 59 | 380 |
| Country Branches (for Tuberculosis) | 120 | 100 | 220 |
| Total ... | 989 | 500 | 1,489 |

10. Of the bed extensions thus passed by the Fund, 1,176 were at hospitals which come within the scope of the Red Cross distribution; but, in view of the decision to allocate to this purpose only a small proportion of the total sum, the number of additional beds taken into account in determining the grants was only 649.

Importance of Proper Housing for Nurses.

11. The amount devoted to schemes other than bed extensions has been used to assist numerous improvements which are no less important from the point of view of the treatment of ex-Service men, and are often of greater urgency. At some hospitals these other improvements are combined with bed extensions; at some hospitals they are the only schemes put forward. They include the enlargement of out-patient accommodation, the improvement or equipment of massage, electrical, and *x*-ray department, new operating theatres, and the provision of better accommodation for nurses. In view of the extent to which the success of medical and surgical treatment depends on nursing, and the importance of housing nurses properly if they are to do their work efficiently, the Distribution Committee had no hesitation in regarding nurses' homes as improvements within the scope of the special distribution.

London Hospitals' Great Benefit.

12. The Distribution Committee would desire to record their sense of the great benefit which the Joint Committee of the British Red Cross Society and the Order of St. John of Jerusalem have conferred on the London Hospitals by placing this large sum at the disposal of the Fund for these purposes. The thorough examination which the Committee have had to make of the schemes submitted has convinced them that, in spite of the present financial difficulties in providing for ordinary maintenance, there are numerous improvements which, having been long deferred through the war, now urgently require attention; and this munificent gift of £250,000 will greatly assist the hospitals in their efforts to remedy the most pressing defects in their accommodation and equipment.

Thanks to King's Fund Secretary.

13. The Committee cannot close their report without placing on record their appreciation of the painstaking and efficient manner in which the heavy work devolving on Mr. Maynard in connection with this special distribution has been carried out.

The list of grants is appended.

For the Committee,

July 1920. John Tweedy, *Chairman*.

**Sir Frederick M. Fry** (Hon. Secretary) presented the list of awards (see p. 458).

**The Royal President's Message.**

**Viscount Finlay,** in moving the adoption of the Report and Awards, said :—

My Lords, Ladies and Gentlemen,—I have first the honour to read the following message from His Royal Highness the Prince of Wales, President of the Fund :—

"I am very sorry to miss the special meeting of the Council of King Edward's Hospital Fund to distribute the £250,000 entrusted to the Fund by the Joint Committee of the British Red Cross Society and the Order of St. John of Jerusalem in aid of schemes of extension or improvement at London hospitals treating ex-sailors or ex-soldiers.

"Please convey my personal thanks to the Joint Committee for this magnificent gift, and to the Distribution Committee for all the extra work it has involved during the past few months.

"I am delighted that the Fund of which I am President should be associated with this scheme for the benefit of ex-Service men, to whom we all owe so much and of whom we are all so proud.

"I hope that this great gift of £250,000 towards urgent improvements will, with the £250,000 just given by the Fund to relieve the most pressing maintenance needs, and other schemes still under discussion, be well backed up by the public and thus go far to assure the safety of our hospital system. "Edward P."

(Hear, hear.)

Distribution Committee's Prompt Action.

This is the second special distribution of £250,000 made through the agency of the Fund this year. On June 22 the Council met and decided to make an emergency distribution of £250,000 out of the capital assets of the Fund to assist the hospitals in their pressing need of funds for current maintenance. The distribution of that sum amongst more than 100 hospitals was carried out with the rapidity appropriate to an emergency, and the cheques were posted by the evening of July 5, less than a fortnight after the original decision was taken; and we are much indebted to the Distribution Committee for the promptness of their action.

Second Distribution of £250,000.

The Council meets again now, on July 23, to carry out another distribution similar in amount but entirely different in its origin, in its objects and in the length of time which it has been necessary for the Distribution Committee

**Viscount Finlay's Speech**—(*continued*).

to spend on it. This second £250,000 does not come from our ordinary assets : it is the gift of the Joint Committee of the British Red Cross Society and the Order of St. John of Jerusalem out of the surplus funds at their disposal at the end of the war, and we are distributing it on their behalf. The objects and conditions are as nearly as possible the same as those of the similar distribution made by the Joint Committee itself amongst provincial hospitals. None of it is to go to maintenance—it is to be used to assist definite schemes of extension or improvement. The distribution is confined on the one hand to hospitals which can and will treat ex-sailors or ex-soldiers; while on the other hand it is extended, at the express wish of the Joint Committee, to some hospitals which come within this condition, but which are not eligible under the rules attaching to grants made out of our general funds. In considering the awards the Distribution Committee have been able to make a thorough examination of the schemes for which applications have been received, and they have devoted many months to this work.

A Much-needed Gift.

This munificent gift of the Joint Committee could not have come at a time when it was more needed or when the Distribution Committee were in a better position to undertake its distribution. Many schemes of extension and improvement have been deferred during the war and had become urgent. Some of these had been submitted to the Fund before the war, and had been examined and reported on by the Distribution Committee after the hospital concerned had been specially visited by a sub-committee. A much larger number were being prepared after the war. A great many of these included the provision of additional beds for in-patients, and the Distribution Committee had announced their intention of making a general survey of all such schemes from the point of view of urgency, geographical position, and prospect of funds not only for the capital expenditure, but also for future maintenance. They were already engaged on this survey when the invitation from the Red Cross Society and the Order of St. John was received. The special distribution has therefore meant, not that they have taken up anything new, but that they have had to concentrate into the last few months the consideration of a great many schemes which would otherwise have come before them at intervals spread over a much longer period. I mention this because it would be a pity if the impression were created that grants were being recommended to schemes which were put forward unnecessarily, and only because it had been announced that this large sum of money was available. The Red Cross Society and the Order of St. John themselves put an initial check on anything of this sort by the requirement that, where a grant is made, an equal sum must be raised by the hospital. And the Distribution Committee, having a very long experience in the examination of schemes of extension or improvement, were able to judge as to their necessity and urgency.

Safeguard for the Hospitals.

The results are summarised in the Report now before us. The Distribution Committee drew a distinction between schemes for the provision of additional beds, and schemes which would not involve any proportionate increase in future maintenance expenditure. In the case of the former, they required full statements as to the reasons for the additional beds, and the prospect of funds for maintenance, and only passed the proposals subject to the proviso that the Hospital Committees were satisfied that they could raise the money both for providing the beds and for keeping them open. You will notice that, out of 3,445 additional beds definitely contemplated, the Fund has so far passed 1,489, postponed 429, and referred back 1,188. These figures include beds at hospitals for women or children as well as at those treating ex-Service men. Taking only the hospitals eligible for Red Cross grants the number of additional beds passed is 1,176. But it did not follow that all of these beds could safely be put in hand in the near future. The Committee, therefore, decided that only a small proportion of the £250,000 should go to bed extensions; and in the end the number of additional beds actually taken into account at the special distribution was only 649. By thus sifting the schemes according to relative urgency, the Distribution Committee have guarded against the danger that the Red Cross money might unduly encourage the hospitals to make themselves liable for increased maintenance expenditure.

Increased Expenditure for Maintenance Discouraged.

The bulk of the £250,000 has thus been allocated to improvements that will not involve any considerable increase in maintenance expenditure. These include enlarged out-patient departments, new operating theatres, improved nurses' quarters, and the equipment of special departments of which so many have been developed in recent years, and are proving so valuable in the treatment of disabled men. The plans and estimates for these schemes, where they are of any size, have also been thoroughly examined and the hospitals visited.

**Hospitals' Debt to the Joint Committee.**

But while we are very grateful to the Distribution Committee for the way in which they have carried out the heavy work involved, we must not for a moment forget that it is to the British Red Cross Society and the Order of St. John that we, and the hospitals, are indebted for the money. It is part of that great fund raised during the war for our sailors and soldiers by the magnificent efforts of the two societies, and by the splendid public response to those efforts. By giving these large sums, out of the surplus left in hand after the war, to the improvement of hospital provision for the treatment of ex-Service men, the Joint Committee both recalled the objects for which the money was subscribed during the war, and foreshadowed that interest in the voluntary civil hospitals in time of peace which has now taken shape in the Hospital Service Department of the Joint Council of the British Red Cross Society and the Order of St. John of Jerusalem, and from which all concerned in hospital work are hoping such great things. You will have an opportunity of formally tendering your thanks to the Joint Committee in the resolution which comes later in the agenda. In the meantime, I would express, on behalf of myself and my colleagues on the President's Powers Committee, our appreciation of their generosity to the London hospitals in giving this large sum of £250,000, and of their action in entrusting this Fund with the distribution. (Hear, hear.) I now beg to move the adoption of the Report.

**The Lord Mayor** (Sir Edward Cooper) having seconded the motion, the adoption of the Report and Awards was put and carried unanimously.

General Council's Thanks.

The Governor of the Bank of England (member of the President's Powers Committee) said that after the observations of Lord Finlay, with which they would all cordially agree (especially the last sentences in regard to

**King's Fund Distribution**—(*continued*).

the generosity of the gift which they were here to distribute), he would content himself with formally moving the following resolution : "That the General Council of King Edward's Hospital Fund tender their best thanks to the Joint Committee of the British Red Cross Society and the Order of St. John of Jerusalem for having set aside the sum of £250,000 out of the surplus funds at their disposal in aid of schemes of extension and improvement at London hospitals treating ex-sailors and ex-soldiers and for entrusting King Edward's Hospital Fund with its distribution."

The resolution, having been seconded by **Lord Stuart of Wortley,** was then put and carried unanimously.

**The Earl of Iveagh** moved a cordial vote of thanks to Viscount Finlay for his kindness in presiding.

The resolution was seconded by the **Chief Rabbi** (the Rev. J. H. Hertz, D.D.) and carried unanimously.

**Viscount Finlay** having briefly acknowledged the vote of thanks, the proceedings terminated.

